# Neonatal Outcomes of Umbilical Cord Milking, Early Cord Clamping and Delayed Cord Clamping in Term Infants: A Randomised Controlled Trial

**DOI:** 10.7759/cureus.78922

**Published:** 2025-02-12

**Authors:** Saifon Chawanpaiboon, Sunisa Nuanjeen, Thitiya Wayuphak, Geeranda Oncharoen, Arunee Phuengphaeng, Julaporn Pooliam

**Affiliations:** 1 Obstetrics and Gynaecology, Faculty of Medicine, Siriraj Hospital, Mahidol University, Bangkok, THA

**Keywords:** delayed cord clamping, early cord clamping, haematocrit, haemoglobin, micro-bilirubin, neonatal anaemia, umbilical cord milking

## Abstract

Introduction

Optimal umbilical cord management at birth plays a crucial role in neonatal outcomes. While delayed cord clamping (DCC) has been associated with improved neonatal haematologic and hemodynamic status, early cord clamping (ECC) remains widely practised. Umbilical cord milking (UCM) has emerged as an alternative to DCC, potentially offering similar benefits while reducing the delay in neonatal resuscitation. This study aims to compare the effects of UCM, ECC, and DCC on neonatal outcomes in term infants born between 37 and 42 weeks of gestation.

Methods

A total of 225 pregnant women at 37-42 weeks’ gestation were randomised into three groups: ECC, UCM, and DCC. Participants delivered by spontaneous vaginal delivery or caesarean section. Newborn haemoglobin, haematocrit, and micro-bilirubin levels were measured within 48 to 72 hours of birth. Maternal demographic data, blood loss volume, newborn Apgar scores at 1 and 5 minutes, neonatal complications, phototherapy requirements, and hospital stay durations were recorded. Baseline maternal characteristics, including antenatal visit counts and haematocrit levels at 32-34 weeks, were similar across groups.

Results

All three groups of pregnant women had no difference in baseline characteristics, number of antenatal visits and baseline haematocrit in the first visit and during 32-34 weeks of gestation. Haemoglobin and haematocrit between the DCC group (17.35 g/dl (2.18), 48.38% (5.76)), the UCM group (17.34 g/dl (2.09), 48.09% (5.70)) and in the ECC group (16.27 g/dl (1.95), 44.92% (5.080)) were different with statistically significant. Other results, including estimated blood loss, neonatal bilirubin, the requirement of phototherapy, blood exchange and neonatal complications, were not different among the three groups.

Conclusion

UCM and DCC improve neonatal haematologic parameters compared to ECC, with DCC showing the greatest benefits. However, DCC is associated with a higher risk of neonatal jaundice requiring phototherapy. UCM may serve as a viable alternative when immediate resuscitation is needed. Further research is required to refine optimal cord management strategies.

## Introduction

The umbilical cord is an important route for nutrient and oxygen transportation from the mother through the placenta and entering to feed the foetus. The umbilical cord consists of two umbilical arteries and one umbilical vein, joined by a mucoid connective tissue called, and covering outside with, Wharton’s jelly. Umbilical arteries carry waste products, including carbon dioxide, via the mother’s circulatory system and dispose of in the lungs along with the mother’s exhaled breath. The veins carry nutrients and oxygen from the mother to the foetus.

For over 60 years, immediate clamping of the umbilical cord was recommended for postpartum haemorrhage (PPH) prevention [1]. At present, we found that early cord clamping (ECC) is related to more adverse neonatal outcomes because the newborn is deprived of the opportunity to receive at least 214 g of blood. The lungs are not fully expanding after immediate delivery [2], resulting in high resistance in the pulmonary arteries with poor heart contraction and neonatal bradycardia. The newborn will have a risk of anaemia at 4 months of age, which results in decreasing motor skills of small muscle movements (i.e., hands and fingers) and social skills at 4 years of age [3].

If there is no urgent obstetric condition in order to prevent bradycardia in newborns, the American Academy of Pediatrics recently recommends clamping the umbilical cord more slowly or waiting until the pulseless of the umbilical cord [4]. Other benefits are increasing blood supply to vital organs such as the brain, heart, lungs and other organs [5]. The total amount of body iron in premature and term newborns will increase up until the age of 6 months without causing harm to the mother and the newborns [6]. By doing delayed clamping of the umbilical cord by 30-180 seconds, the blood volume will increase by 80-100 cc, and blood iron will increase by about 40-50 mg/kg or 115-125 mg in full-term infants [7]. This will help reduce iron deficiency anaemia in the first year of life and improve growth and emotional development. The amount of stem cells will increase and result in improving the immune system [7]. Other benefits of delay cord clamping (DCC) include improving the circulatory system in the newborn, elevating blood pressure, reducing oxygen and ventilator usage and increasing blood volume. The risks of intravenous fluid transfusion, inotropic drug administration, intraventricular haemorrhage (IVH), necrotising enterocolitis and infection will be reduced. Overall, the development of the nervous system will be improved [8].

Physiologic anaemia in infancy occurs mostly around 12 weeks after birth [9]. Preterm and low birthweight newborns with birthweights of only 1-1.5 kg have a risk of anaemia (haemoglobin of 7-8 mg/dL) immediately after birth. If the newborn receives an increased amount of blood immediately after birth, the risk of anaemia can be reduced during the postpartum period and up to 12 weeks of age [9].

However, DCC is not feasible if the baby has extremely low birth weight [10], a condition that requires urgent postpartum support or maternal complications during the postpartum period (i.e., placenta previa, placenta abruption) [11]. DCC may be associated with hypothermia, hyperbilirubinemia, polycythaemia and transient tachypnoea in newborns without being associated with other neonatal diseases or PPH [12]. Therefore, umbilical cord milking (UCM), which usually takes less than 5 seconds, is one way to increase blood volume in newborns immediately. UCM also reduces anaemia in newborns [13].

Our study aims to compare the effects of ECC, DCC, and UCM to discern any differences in outcomes. The rationale for this investigation is bolstered by recent guidelines recommending DCC, underscoring the importance of evaluating its comparative efficacy and safety in clinical practice.

## Materials and methods

This prospective, randomised controlled trial was conducted in the labour and operating room of Siriraj Hospital from 1st January 2021 to 31st March 2022. The study protocol was approved by the Siriraj Institutional Review Board, Faculty of Medicine, Siriraj Hospital, Mahidol University (Certificate of approval no. Si 822/2020). The trial was registered with the Thai Clinical Trials Registry (TCTR) under the registration number TCTR016431006.

The main objective was to examine the blood concentrations (haemoglobin and haematocrit) in newborns within 48-72 hours after birth across three groups. The secondary objectives included assessing bilirubin levels in newborns, neonatal hypothermia, and postpartum blood loss among the three groups. Pregnant women who delivered during the gestational age of 37-42 weeks and consented to participate in the research were included. The exclusion criteria included pregnant women under 18 years of age, short umbilical cord (less than 25 cm long), abnormal umbilical cord, pregnant women with obstetric emergencies and the need for immediate delivery, such as prolapse cord, preterm placenta ablation, placenta previa, pre-eclampsia, uterine rupture, etc., pregnant women with severe blood loss, shock (hemodynamic unstable), twin pregnancies, pregnant women with HIV, syphilis or hepatitis B, Rh negative blood results and newborns with abnormalities, including congenital malformations, oedema, foetal growth restriction, prenatal stress or irregular heartbeat (NICHD (National Institute of Child Health and Human Development) category 2 or 3 foetal distress), or those not breathing or crying at birth and requiring resuscitation per Neonatal Resuscitation Program (NRP) 2020 guidelines.

**Figure 1:**
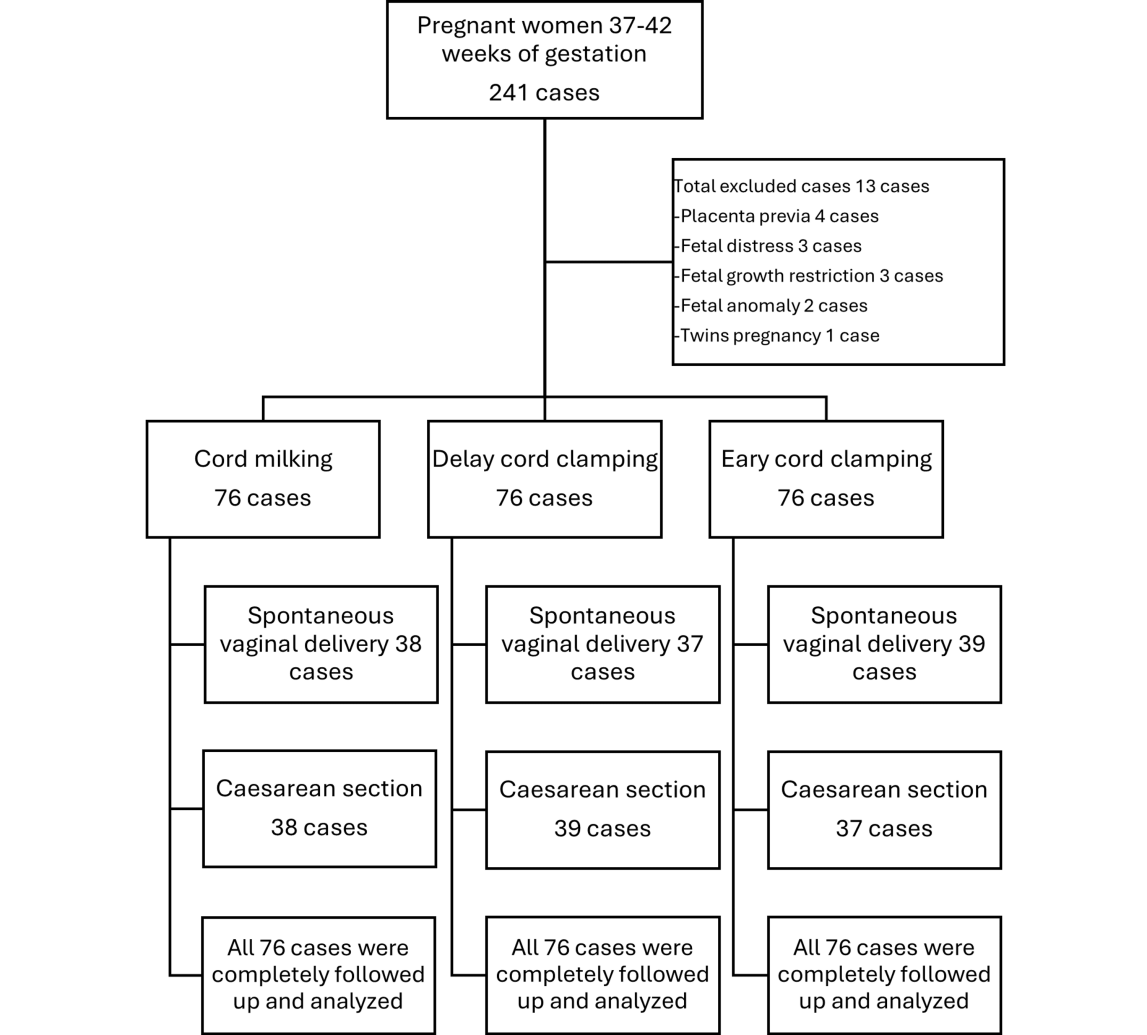
Diagram illustrating the recruitment process of participants The image was created by Saifon Chawanpaiboon (One of the authors of this article).

This research was conducted by recruiting 228 pregnant women with a gestational age of 37-42 weeks delivered by spontaneous vaginal delivery and caesarean section. They were advised and signed an acknowledgement of participation form and then randomly assigned into three groups of study (76 women in each group): UCM, DCC, and ECC by block randomisation. (Figure 1). We used a computer-generated randomisation sequence to allocate participants into the three study groups. The Sequentially Numbered, Opaque, Sealed Envelope (SNOSE) method was used to ensure allocation concealment. Randomisation was performed in blocks of six to maintain balanced group sizes throughout the study.

The baby delivered by caesarean section was placed on the front of the mother’s legs, and the baby delivered by spontaneous vaginal delivery was placed on the mother’s abdomen.

UCM was performed by pumping blood in the umbilical cord from the maternal side to the baby starting at 25 cm from the baby’s abdomen, three times at a speed of 10 cm per minute with 2 seconds apart. The umbilical cord was cut at 2-3 cm. away from the baby’s abdomen. [14] In the DCC group, the umbilical cord was clamped at 60 seconds after birth and cut 2-3 cm from the infant’s abdomen [15]. ECC was performed by clamping the cord less than 30 seconds after delivery. The blood concentration and micro-bilirubin within 48 and 72 hours were tested.

This study was conducted as an open-label, non-blinded, randomised controlled trial. Due to the nature of the intervention, blinding of clinicians and caregivers was not feasible. However, outcome assessors evaluating neonatal parameters, including Apgar scores, neonatal complications, need for phototherapy, and length of hospital stay were blinded to the intervention group to minimise bias. Demographic data, maternal blood loss, and neonatal outcomes were systematically recorded.

Demographic data, the amount of maternal blood loss, Apgar scores of newborn babies at 1 and 5 minutes, neonatal complications, phototherapy and length of hospital stays were recorded.

Definition

PPH is defined as blood loss exceeding 1000 cc [16]. Bowel ileus is a condition where the bowel malfunctions without any underlying structural abnormality [17]. Postnatal adaptation refers to the transition to extrauterine life, marked by changes in circulatory pathways, the initiation of lung ventilation and oxygenation, and various metabolic adjustments [18]. Polycythaemia in newborns is defined as a central venous haematocrit level exceeding 65% [19]. Transient tachypnoea of the newborn is a respiratory condition that occurs shortly after birth, caused by retained foetal lung fluid due to impaired clearance mechanisms [20].

Sample size calculation

According to data from a research study by Panburana et al. [21] found that the haemoglobin concentration in the UCM group was 17.0 g/dl (standard deviation (SD)=1.9), and the DCC group was 16.5 (SD=1.6). The investigator assigned a clinically significant difference equal to 1 g/dl [15]. Therefore, when the statistical significance level was 0.05 (type I error = 5%, 2-sided) and the power of the test = 90% (type II error = 10%). The sample size must be 66 people per group. Because in this study, there was a control group, namely the normal postpartum umbilical cord-cutting group, therefore, the number of samples in each group was 66 cases. When a 10% loss to follow-up or withdrawal from the study was calculated, a total of 228 participants with 76 cases per group were needed.

Statistical analysis

The baseline characteristics, as well as the primary and secondary outcomes, were investigated. Differences between groups were examined using a one-way analysis of variance (ANOVA) for continuous data and a chi-square test for categorical data. Outcomes between groups were analysed by using one-way ANOVA for normally distributed continuous data, Kruskal Wallis test for non-normally distributed continuous data, and chi-square test for categorical data. Differences between groups were examined using the student t-test (for normal distribution) or Mann-Whitney U test (for non-normal distribution) for continuous data. Pearson’s chi-square test was used to analyse categorical data. Categorical data are expressed as a number (percentage) and as a mean (interquartile range; IQR) for continuous data. Normally distributed data are expressed as a mean (SD). The P-value of the 2-sided proportion difference is provided for all outcomes, with 0.05 considered significant. All analyses were based on the assigned group and completed with IBM Statistical Package for Social Sciences (SPSS) version 26.0 software (SPSS Inc., Chicago, IL, USA).

## Results

Between 1 January 2021 and 31 March 2022, women who were expected to deliver between 37 and 42 weeks of gestation at Siriraj Hospital were approached. In all, 228 eligible women agreed to be enrolled in the study. They were assigned to the three study groups.

The baseline characteristics, underlying diseases, and haematocrit levels among the three groups (UCM, DCC, and ECC) were generally comparable. Maternal age, body weight, and body mass index showed no significant differences among groups (P = 0.622, 0.789, and 0.618, respectively). Similarly, occupation and income distribution were not significantly different (P = 0.654 and P = 0.462, respectively). Obstetric history, including first parity and parity >1, also showed no significant variations (P = 0.361 and P = 0.330, respectively). However, gestational age at delivery was significantly different among groups (P = 0.045), with the ECC group delivering at a slightly later gestational age (mean 38.7 weeks) compared to the DCC (38.2 weeks) and UCM (37.2 weeks) groups. Antenatal care visits also showed a significant difference (P = 0.033), with a higher proportion of participants in the UCM group attending more than 10 visits. Although underlying diseases were not significantly different among groups (P = 0.063), diabetes mellitus was more prevalent in the DCC group (11.9%) compared to the UCM (6.6%) and ECC (1.3%) groups. Haematocrit levels in the first trimester and at 32-34 weeks of gestation did not significantly differ among the groups (P = 0.624 and P = 0.117, respectively) (Table 1). No instances of PPH were recorded in any of the groups (UCM, ECC, DCC).

**Table 1:** Baseline characteristic data, underlying diseases and haematocrit levels at the first trimester and 34 weeks of gestation One-way analysis of variance (ANOVA) for continuous data and the chi-squared test for categorical data. ANC: antenatal care; GA: gestational age The “±” symbol as representing the mean ± standard deviation (SD). The superscript asterisk (*) indicates statistical significance at P < 0.05.

Variable	Umbilical cord milking (N=76)	Delayed cord clamping (N=76)	Early cord clamping (N=76)	P-value
Age (yr)	33.0±4.9	33.7±5.5	32.0±5.8	0.622
Body weight (kg)	70.9 ±12.3	72.4±12.6	71.6±14.0	0.789
Body mass index (kg/m^2^)	27.7±4.6	28.3±4.6	28.4±5.1	0.618
Occupation				0.654
Housewife	9 (11.8%)	14 (18.4%)	16 (21.0%)	
Employee	33 (43.4%)	33 (43.4%)	39 (51.4%)	
Government	26 (34.3%)	20 (26.4%)	15 (19.7%)	
Merchant	3 (3.9%)	3 (3.9%)	3 (3.9%)	
Personal business	3 (3.9%)	3 (3.9%)	2 (2.6%)	
Student	2 (2.7%)	3 (3.9%)	1 (1.3%)	
Income (Baht/month)				0.462
< 10 000	3 (3.9%)	4 (5.3%)	7 (9.2%)	
10 000–19 999	14 (18.4%)	11 (14.5%)	15 (19.7%)	
20 000–49 999	24 (31.6%)	33 (43.4%)	28 (36.8%)	
≥ 50,000	35 (46.1%)	28 (36.8%)	26 (34.2%)	
First parity	37 (48.7%)	32 (42.1%)	29 (38.1%)	0.361
Parity > 1	31 (40.8%)	37 (48.7%)	40 (52.6%)	0.330
GA at delivery (weeks) (Mean (min, max))	37.2 (37,40)	38.2 (37, 41)	38.7 (37, 41)	0.045
ANC (number of visits)				0.033
< 5	3 (3.9%)	4 (5.3%)	7 (9.2%)	
5–10	52 (68.4%)	57 (75.0%)	57 (75.0%)	
> 10	21 (27.7%)	19 (25.0%)	12 (15.8%)	
Underlying disease				0.063
None	64 (84.2%)	63 (82.9%)	72 (94.7%)	
Diabetes mellitus	5 (6.6%)	9 (11.9%)	1 (1.3%)	
Hypertension	1 (1.3%)	2 (2.6%)	0 (0.0%)	
Other	6 (7.9%)	2 (2.6%)	3 (3.9%)	
Haematocrit in the first trimester	37.2±2.6	37.02±3.2	36.65±3.5	0.624
Haematocrit at 32–34 weeks of gestation	36.35±2.2	36.08±3.1	35.35±3.6	0.117

Regarding neonatal outcomes, haemoglobin and haematocrit levels at birth were significantly higher in the UCM and DCC groups compared to the ECC group (P = 0.001 and P < 0.001, respectively). There were statistically significant differences between the neonatal haemoglobin and haematocrit levels of the ECC (control) group (16.27 g/dl (1.95); 44.92% (5.080)), the UCM group (17.34 g/dl (2.09); 48.09% (5.70)) and the DCC group (17.35 g/dl (2.18); 48.38% (5.76); Table 2). There were no instances of hypothermia observed in the newborns across the three groups.

However, micro-bilirubin levels did not significantly differ among groups (P = 0.401). The mode of delivery and estimated blood loss were similar across groups (P = 0.986 and P = 0.724, respectively). Birth weight, Apgar scores at 1 and 5 minutes, and neonatal sex distribution showed no significant differences (P = 0.706, P = 0.288, and P = 0.854, respectively). Neonatal complications, including transient tachypnoea of the newborn, postnatal adaptation issues, polycythaemia, and bowel ileus, were not significantly different among groups (P = 0.821). Phototherapy use was highest in the UCM (13.2%) but was not statistically significant (P = 0.129). The duration of hospital stay was similar across groups (P = 0.136. Overall, while cord management strategies significantly impacted haemoglobin and haematocrit levels, other neonatal outcomes remained comparable across the groups (Table 2).

**Table 2:** Comparison of the outcomes of the three groups Independent t-test for normally distributed continuous data, the Mann–Whitney U test for non-normally distributed continuous data, and the chi-squared test for categorical data. TTNB: transient tachypnoea of the newborn The “±” symbol as representing the mean ± standard deviation (SD). The superscript asterisk (*) indicates statistical significance at P < 0.05.

	Umbilical cord milking (N=76)	Delayed cord clamping (N=76)	Early cord clamping (N=76)	P-value
Primary outcome				
Haemoglobin	17.3 ±2.2	17.3±2.1	16.3±1.9	0.001^*^
Haematocrit	48.4±5.8	48.1±5.7	44.9±5.1	< 0.001^*^
Secondary outcome				
Micro-bilirubin	8.7±1.7	8.7±2.2	8.3±1.5	0.401
Delivery				0.986
Spontaneous	38 (50.0%)	37 (48.7%)	39 (51.3%)	
Caesarean section	38 (50.0%)	39 (51.3%)	37 (48.7%)	
Blood loss (Mean (min, max))	300 (100, 1600)	300 (50, 1500)	300 (50, 1000)	0.724
Birth weight (g)	3082.8±392.8	3080.1±300.0	3121.3±320.9	0.706
Apgar score				
at 1 minute	8.6±0.7	8.5±0.8	8.4±0.8	0.288
at 5 minutes	9.6±0.5	9.4±0.6	9.6±0.5	0.054
Sex				0.854
Male	37 (48.7%)	37 (48.7%)	40 (52.6%)	
Female	39 (51.3%)	39 (51.3%)	36 (47.4%)	
Neonatal complication	5 (6.6%)	5 (6.6%)	3 (3.9%)	0.821
TTNB	1	3	1	
Postnatal adaptation	1	0	2	
Polycythaemia	0	1	0	
Bowel ileus	1	0	0	
Phototherapy	10 (13.2%)	8 (10.5%)	3 (3.9%)	0.129
Duration of admission (Days) (Median (min, max))	3 (2, 8)	3 (1, 8)	3 (2, 8)	0.136

## Discussion

Our study compared the effects of UCM, DCC, and ECC on neonatal outcomes. While maternal characteristics were generally comparable across groups, gestational age at delivery was significantly lower in the UCM group (p=0.045). Notably, haemoglobin and haematocrit levels were significantly higher in the UCM and DCC groups than in the ECC group (p=0.001 and p<0.001, respectively), suggesting improved neonatal haematologic status. However, secondary outcomes, including micro-bilirubin levels, birth weight, Apgar scores, neonatal complications, need for phototherapy, and duration of hospital admission did not differ significantly between groups. These findings indicate that UCM and DCC may enhance neonatal haematologic parameters without increasing adverse outcomes.

DCC and UCM resulted in more blood flow transferring to newborns than ECC. Both methods of DCC and UCM had similar results of increasing haemoglobin and haematocrit in newborns [22]. However, the superior benefit of UCM compared to DCC is the shortening duration period from delivery to initial resuscitation by a paediatrician. UCM has been shown to be feasible in infants requiring resuscitation at birth. DCC is recommended for all premature births despite some studies suggesting a decreased placental transfusion at caesarean delivery. UCM from the placental side towards the newborn is an alternative to placental transfusion from DCC. [2] UCM has been shown to improve haemoglobin levels and short-term clinical outcomes in preterm infants.

Hypothermia, over-transfusion, jaundice, polycythaemia, persistent pulmonary hypertension, and delays in resuscitation efforts may be the potential risks associated with DCC. However, recent research indicates that there is no increased risk of hyperbilirubinemia and increased admission to the neonatal intensive care unit with DCC. A recent study showed that hypothermia may not be a significant risk of delay cord clamping [23], and hypothermia was not found in our study.

From recent evidence, DCC in term and preterm infants is related to higher haemoglobin levels, improvement of children’s neurodevelopment, low incidence of anaemia, higher blood pressure, fewer transfusions, decreased incidence of IVH, chronic lung disease, necrotising enterocolitis, and late-onset sepsis. DCC also had a low incidence of neonatal hypothermia, respiratory distress, severe jaundice, PPH and maternal blood transfusion, both delivery by caesarean section or vaginal delivery [6]. DCC may lead to excessive blood transferring to the foetus, leading to polycythaemia, but our studies did not find polycythaemia in all newborns.

The 2022 intrapartum care guidelines of the National Institute for Health and Clinical Excellence (NICE) [24] recommended that DCC prevent PPH by using effective prevention methods. Other prevention methods are administering uterotonic agents, controlling umbilical cord traction, and uterine massage [23,25]. DCC significantly increases the levels of haemoglobin, Serum ferritin and haematocrit at 4 weeks of age. It should be recommended in routine practice where it is not contraindicated, especially in resource-poor settings [26].

DCC for at least three minutes is recommended. The risk of DCC may increase the risk of PPH and hypothermia, but our research did not find both conditions. The newborn’s iron reserves are increased. Many studies supported the reduction of anaemia in the first month after birth [26] and may improve the long-term infants’ neurological outcomes [27].

Based on our findings, we recommend delaying umbilical cord clamping for 60 seconds after birth. We continue to support the American College of Obstetricians and Gynecologists (ACOG) recommendations [28] due to the robust evidence from large trials. However, there are several limitations in our study. First, the sample size may not have been large enough to detect rare neonatal complications associated with different cord clamping strategies. Second, although our findings support the benefits of DCC, we limited the delay to 60 seconds, whereas the ACOG guidelines recommend a delay of at least 30-60 seconds. Third, our study did not assess long-term neonatal outcomes, which could offer valuable insights into the benefits and risks of DCC. Finally, while we excluded newborns requiring immediate resuscitation, future research should explore how DCC can be safely implemented in this population. DCC increases a newborn’s iron reserves, with many studies reporting reductions in anaemia during the first month after birth [26].

Although we did not conduct detailed subgroup analyses, we acknowledge the importance of such analyses in identifying populations that may benefit more from one cord-clamping method over another. For example, some studies have raised concerns about PPH and delayed resuscitation in the context of DCC, but our findings suggest that these concerns may not be as significant in practice, as no PPH was observed in our cohort. Future studies with larger sample sizes and more granular subgroup analysis are needed to further elucidate these outcomes, particularly with regard to neonates requiring immediate resuscitation at birth.

DCC may also improve an infant’s long-term neurological outcomes [27]. Although there is a risk that DCC may increase the risk of PPH or hypothermia, our research did not find either condition. The World Health Organization recommends DCC for term infants. DCC in term infants increases haemoglobin levels at birth and improves iron stores in the first later months of life, which may benefit developmental outcomes [28]. The incidence of jaundice requiring phototherapy in term infants is slightly increased [28].

Furthermore, even though UCM was found to reduce the need for blood transfusions, the procedure did not improve clinical outcomes [29]. Further research is needed to confirm these adverse outcomes.

The strengths of the study

This study utilised a randomised design to directly compare the effects of DCC, UCM, and ECC, contributing valuable evidence to optimise blood transfer to newborns. The inclusion of a robust sample size enhanced the reliability and generalizability of the findings, ensuring the results are applicable to a broad clinical population. Furthermore, the study provided clear and actionable recommendations, emphasising the benefits of DCC while highlighting UCM as a viable alternative in specific scenarios. These strengths make the study a significant contribution to evidence-based neonatal care and support informed clinical decision-making.

The limitations of the study

One limitation of this study is that it primarily focuses on the potential benefits of DCC and UCM in non-urgent cases where resuscitation is not immediately required. In newborns requiring immediate resuscitation (such as those not crying at birth), ECC remains necessary to facilitate prompt intervention. As such, the findings may not be directly applicable to these urgent cases. Additionally, while the study supports the advantages of DCC and UCM in improving neonatal outcomes such as haemoglobin and haematocrit levels, the potential risks associated with these interventions - such as hypothermia, jaundice, and polycythaemia - were not observed in this study, and further research is needed to confirm long-term safety. Lastly, our study was not designed to address the impact of DCC and UCM on neurodevelopmental outcomes, and this remains an area for future exploration.

## Conclusions

Our study supports the practice of DCC as a beneficial intervention for improving neonatal outcomes, particularly in preterm infants, by promoting better blood transfer and improving immediate markers such as haemoglobin and haematocrit levels. Given the advantages observed in our study, we recommend that DCC be considered as the standard practice for preterm infants, provided it is clinically feasible.

In cases where DCC is not feasible, UCM presents a viable alternative, offering similar immediate benefits without the need for prolonged delays. Our findings do not include long-term neurodevelopmental assessments, and therefore, future research should explore the potential long-term effects of these interventions.

Further studies are needed to fully understand the broader implications of these practices, including any potential risks, such as the effect of UCM or DCC on conditions like polycythaemia and persistent pulmonary hypertension, particularly in the context of different delivery settings.
